# Understanding and Use of Nutrition Labels of Prepackaged Food by University Students: A Cross-Sectional Study in Chongqing, China

**DOI:** 10.3390/nu14194189

**Published:** 2022-10-08

**Authors:** Hao Wei, Ke Jiang, Boya Liu, Zhichuan Hu, Yong Zhao, Hong Xu, Manoj Sharma, Chuan Pu

**Affiliations:** 1 School of Public Health, Chongqing Medical University, Chongqing 400016, China; 2 Research Center for Medicine and Social Development, Chongqing Medical University, Chongqing 400016, China; 3 Collaborative Innovation Center for Early Warning of Health-Related Major Social Risks, Chongqing Medical University Sub Center, Chongqing 400016, China; 4 Research Center for Public Health Security, Chongqing Medical University, Chongqing 400016, China; 5 Chongqing Key Laboratory of Child Nutrition and Health, Children’s Hospital of Chongqing Medical University, Chongqing 400014, China; 6 Department of Social and Behavioral Health, University of Nevada, Las Vegas, NV 89119, USA

**Keywords:** prepackaged food, food nutrition label, Chongqing, university student, nutrition survey

## Abstract

Object: The correct use of nutrition labels for prepackaged food helps university students develop healthy eating habits and prevent the occurrence of chronic non-communicable diseases. This study evaluates the understanding and use of nutrition labels of prepackaged food by university students in four different fields of study in Chongqing, China. Methods: This cross-sectional study used an online survey platform conducted in July 2022 in colleges and universities in Chongqing, China. The convenience sampling method was used to select students in four different fields of study, including medicine, humanities, science and technology, and arts and sports. Ten questions were used to assess participants’ understanding of nutrition labels. A score of six or above (60%) indicates that the respondent has a basic understanding of the nutritional labels of prepackaged food. Descriptive statistics and generalized linear models (GLMs) were used to assess participants’ understanding and use of nutrition labels for prepackaged foods and their influencing factors. Results: A total of 1262 valid questionnaires was collected. The average age of the participants was 21.8 years (SD: 2.43), 50.1% were male, 80.8% were ethnic Han, and 50.9% were from rural areas. Only 21.3% of the university students in Chongqing had a good understanding of the nutrition labels of prepackaged food, where medical students were the highest (39.9%) and science and engineering students were the lowest (15.6%). Gender, ethnicity, grade, major, and whether received courses related to nutrition were influential factors in the understanding and use of nutrition labels of prepackaged food. Medical students also had more positive attitudes toward nutritional labels of prepackaged food. Conclusions: Understanding and use of nutrition labels for prepackaged food by university students in Chongqing are unsatisfactory. Student’s major was a significant influencing factor in nutrition label comprehension, with medical students having the greatest understanding. Based on these results, we suggest that nutrition and health courses should be popularized among non-medical students to narrow the differences between different fields of study. For university students in all fields of study, education and publicity of nutrition labels of prepackaged food are needed, not only in the classroom but also in daily life.

## 1. Introduction

With the rapid development of the national economy and lifestyle changes, chronic non-communicable diseases (NCDs) have become the main cause of death in China, accounting for 82%, and this proportion will continue to increase [1]. However, diet is an important factor affecting the development of premature death, disability, and chronic diseases [2,3]. Therefore, there is a need to actively improve our diet to control the occurrence of chronic diseases. Among them, nutrition labeling is an effective way to improve people’s eating habits. These results show that people who read nutrition labels eat healthier food than those otherwise [4,5].

Almost everyone chooses prepackaged food when shopping, including university students. The nutrition label on prepackaged food is important for people to obtain the most accurate nutrition information and correctly guide them to choose healthier and more nutritious food [6], including the nutrition composition table, nutrition claim, and nutrition function claim of food. The general rules for nutrition labeling of prepackaged food issued in China stipulate that, for example, prepackaged food produced after 1 January 2013 must be labeled with four nutrients and energy (4 + 1), as well as the percentage of the nutrient reference value (NRV%). Among them, the four nutrients are protein, fat, carbohydrate, and sodium. The rules also make requirements for the format of the nutrition label. In general, the label must be presented in the form of a square table entitled “Nutrition Table” [7] (Supplementary Figure S1). Recently, the Chinese Nutrition Society also revised the 2022 edition of dietary guidelines for Chinese residents and proposed to “be able to cook food scientifically, select and match ingredients correctly, and understand information on nutrition label clearly”. These policies had a positive effect on consumers, which encourage consumers to better control the intake of sugar, fat, low-density cholesterol, etc. [8,9,10], and may reduce the risk of obesity, hyperlipidemia, diabetes, cardiovascular disease, and other nutrition-related chronic non-communicable diseases [10,11,12].

Consumers’ correct understanding and use of food nutrition labels are important prerequisites for obtaining a healthy diet. Studies have shown that there are health-related differences, including attitudinal differences and behavioral differences, between people who regularly read nutrition labels on prepackaged foods and those who do not [13,14]. Attitudinal differences, such as people who read nutrition labels regularly, place more importance on health and healthy eating than those who do not [13]. Behavioral differences are that people who read nutrition labels regularly eat more healthily than those who do not [14].

In addition, people’s understanding and use of the nutrition labels on prepackaged food are also affected by nationality, place of residence, gender, occupation, income level, education, and other factors [7,15,16]. Although a study has shown that field of study is an important factor influencing college students’ use of prepackaged food nutrition labels [16], there is no published literature discussing the influence of field of study on the use of prepackaged food nutrition labels by Chinese college students. Furthermore, it is unclear what factors will affect the understanding and use of university students on food nutrition labels in Chongqing, China. Therefore, this study aimed to evaluate the understanding and use of nutrition labels of prepackaged food by university students in four different fields of study in Chongqing, China, and to explore the influencing factors of university students’ understanding and use of nutrition labels on prepackaged food.

## 2. Methods

### 2.1. Study Design and Sampling

This is a cross-sectional study conducted on the online survey platform “Questionnaire Star” which is a professional online survey platform in China [17]. Convenience sampling method was used to select students from 65 of the 70 universities in Chongqing. We relied on the Chongqing Municipal Commission of Education to collect all the university information in Chongqing. Then, the questionnaire link or QR code was sent to each school’s WeChat group which is a popular instant messenger platform. The administrator of each WeChat group guided the students to complete the questionnaire namelessly and independently. Free, prior and informed consent were extracted from the participants and were asked to answer the questionnaire anonymously. Ultimately, 1262 students from 65 colleges or universities were investigated after excluding outliers and missing values.

### 2.2. Instrument

The questionnaire was designed based on two documents: the General Guidelines on Nutrition Labelling of Prepackaged Foods [18] and the General Guidelines on Nutrition Labelling of Prepackaged Foods (GB 28050-2011) Questions and Answers (Revised Version) [19] both issued by the National Health Commission of the People’s Republic China. The questionnaire consisted of three components: demographic characteristics, knowledge, and attitudes towards nutritional labels of prepackaged food (Supplementary Table S1). To make the options and questions in the questionnaire as accurate and complete as possible, professors of epidemiology, medicine, statistics, and nutrition participated in the development of the questionnaire to help ensure it was accurate and adequately achieved the study objective. 

Demographic characteristics consisted of 12 items: age, biological sex (male vs. female), ethnicity (Han vs. minority), residence (rural vs. urban), height, weight, mother’s education (elementary school and below, junior high school, senior high school, junior college, undergraduate, master or above), father’s education (ibid), per capita monthly household income (<3000, 3000–5000, 5000–10,000, >10,000), caregivers (parents vs. grandparents and others), grade level (first, second, third, fourth, fifth, postgraduate), and whether they received courses related to nutrition (yes vs. no).

Knowledge of nutritional labels on prepackaged food consisted of ten items: prior knowledge of the nutrition label on prepackaged food, frequency of observing food nutrition labels, main contents of the food nutrition label, indicators that must be included in the nutrition label on prepackaged food, prepackaged food that is mandatorily required by the state to label food nutrition, common units of measurement in nutrition labels, the meaning of NRV in the food nutrition label, nutrition labeling specifications for foods with nutrition claims, understanding of the claim of food nutritional function, and identifying the nutritional level of food through food labels.

Attitudes towards nutritional labels on prepackaged food consisted of four items: the need for food nutrition labeling on food bags, whether one trusts the information in the food nutrition label on the food bag, the main purpose of looking at nutrition labels, and main reasons that cause you to read nutrition labels.

Self-reported height and weight were used to compute the body mass index (BMI, weight/height^2^), which was then classified into four categories: underweight (<18.5 kg/m^2^), normal (18.5 kg/m^2^ ≤ BMI < 24 kg/m^2^), overweight (24 kg/m^2^ ≤ BMI < 28 kg/m^2^), and obese (BMI ≥ 28 kg/m^2^) [20]. Father and mother’s education was divided into three categories: low (junior high school and below), medium (senior high school/junior college), and high (college/bachelor’s degree and above). The grade was classified as either low (first and second year of university) or medium (third and fourth year of university or fifth year of university if applicable). Only knowledge of nutritional labels on prepackaged food was calculated as a score, the attitude was not. Correct answers receive one point, while incorrect ones receive none. The total scores for the questions ranged from 0 to 10. A score of six or above (60%) indicates that the respondent has a basic understanding of the nutritional labels on prepackaged food, with a higher score indicating better understanding.

### 2.3. Statistical Analysis

Descriptive statistics were used for the sample characteristics. For categorical variables, frequency and proportions (%) were used, while for continuous variables, mean and standard deviation (SD) were utilized. χ2 tests were used to compare various majors’ knowledge and attitudes towards nutritional labels on prepackaged food, as well as other socioeconomic and demographic factors. To determine if the relationships between the demographic characteristics and understanding and using the nutritional labels on prepackaged food existed, the odds ratio (OR) and 95% confidence interval (CI) for the outcome variable were calculated using generalized linear models after adjusting for residence, gender, ethnicity, parents’ education, caregivers, BMI, income, grade, major, and received courses related to nutrition (when exploring the relationship between one demographic factor and understanding and using the nutritional labels on prepackaged food, the others were adjusted). STATA version 17.0 (STATA Corporation, College Station, TX, USA) was used for all analyses. Statistical significance was considered when *p* < 0.05 (two-sided).

## 3. Results

The sample characteristics of the participants are presented in Table 1. The average age of the entire respondent population was 21.8 (SD: 2.43), where 50.1% were male. Most participants were Han (80.8%), from rural areas (50.9%), with a high level of father’s education (42.7%), a low level of mother’s education (41.9%), per capita monthly household income of 3000 to 5000 (40.9%), raised by grandparents or others (51.0%), and received courses related to nutrition (52.1%). Moreover, 34.1% of participants had abnormal BMI (underweight: 2.5%, overweight: 31.4%, obese: 0.2%). As for the grade of the participants, respondents with low, high, and postgraduate accounted for 32.6%, 37.1%, and 30.3%, respectively.

The responses of students from different fields of study to each question are shown in Table 2. Overall, medical students had the highest pass rate (39.9%) and science and engineering students had the lowest pass rate (15.6%). Specifically, 70.7% of the students knew the nutrition label on prepackaged food and the awareness rate of medical students was the highest (89.3%), while the awareness rate of science and engineering students was the lowest (64.0%). Most students (74.0%) were able to frequently observe food nutrition labels when purchasing prepackaged foods and the frequency was higher for medical students, reaching 83.3%. One-third of students knew what the main contents of food nutrition labels contain and what indicators must be included in the nutrition label on prepackaged food. The question of which prepackaged foods are required by the state to have nutrition labels was poorly answered by students of all majors (19.7%). In addition, 21.2% of the students were able to correctly answer the meaning of NRV in the food nutrition label and the accuracy rate of medical students was 51.8%, higher than that of liberal arts students (16.4%), science and engineering students (27.8%), and art and sports students (26.7%). Moreover, a total understanding of the claim of food nutritional functions was found in 32.2% of the students, of which medical students were the highest (56.5%) and liberal arts students were lowest (26.2%). Medical students had the highest accuracy rate (37.5%) and art and sports students had the lowest accuracy rate (20.7%) in identifying the nutritional level of food through food labels.

The results of the generalized linear model are shown in Table 3. After adjusting for other confounding factors, females (OR: 1.26 CI: 1.03–1.55) were more likely to understand nutritional labels on prepackaged food. Compared with junior university participants, senior (OR: 1.36 CI: 1.05–1.78) and graduate (OR:1.35 CI: 1.02–1.78) university participants had a high probability of understanding of nutritional labels on prepackaged food. Those who were minorities (OR: 0.10 CI: 0.04–0.25), with per capita monthly household income > 10,000 (OR: 0.51 CI: 0.27–0.94), majoring in liberal arts (OR: 0.62 CI: 0.47–0.81), science and engineering (OR: 0.58 CI: 0.43–0.79), or arts and sports (OR: 0.68 CI: 0.50–0.93) and had not received courses related to nutrition (OR: 0.57 CI: 0.45–0.73) were less likely to have understood nutritional labels on prepackaged food, compared with those who were ethnic Han, with per capita monthly household income < 3000, majoring in medicine, and had received courses related to nutrition.

Attitudes towards nutritional labels on prepackaged food are shown in Table 4. Regardless of the major, most students believed that food bags need to be labeled with food nutrition labels (71.9%), but only 4% thought it does not matter whether these are labeled or not (*p* = 0.14). Only 20.4% of participants fully trusted the information on the food nutrition label on the food bag and 16.8% of the participants did not trust the information on the food nutrition label on the food bag at all (*p* = 0.052). On using food nutrition labels, the students who blindly and casually observed were the highest (35.2%) and the students of medicine were the least (12.5%) (*p* < 0.001). When discussing the frequency of using food nutrition labels among those who use them, medical students were the highest (22.6%) and art and sports students the lowest (4.7%) (*p* < 0.001).

## 4. Discussion

With the release of dietary guidelines for Chinese residents (2022) on 26 April 2022, we conducted a cross-sectional survey on the status of knowledge of nutrition labels on prepackaged food among university students in Chongqing, China. This cross-sectional study was designed to evaluate the understanding and use of nutrition labels for prepackaged food by university students in Chongqing, China. The overall results showed that the understanding and use of food nutrition labels among university students in Chongqing was poor: only 21.3% of university students reported that they understood prepackaged food nutrition labels, 48.4% of university students often or always use nutrition labels, and the performance of medical students was better than that of non-medical ones. Another study in Shanghai, China, showed that only 35.3% of parents of students knew nutrition labels and the utilization rate was only 19.3% [21]. However, a study in Zimbabwe showed that 40.9% of urban and rural adults in Zimbabwe understood the nutrition label on prepackaged food [13]. Another study in Iran reported that 47.6% of students often or always use nutrition labels and 32.3% integrate the information of food nutrition labels into their daily diet [17]. Another study in South Korea reported that more than 70% of consumers across Korea can easily understand food nutrition labels and 42.2% of consumers often use food nutrition labels [22]. Summarily, university students in Chongqing have a poor understanding and use of nutrition labels for prepackaged food compared with foreign countries. 

Through our research, we found that the number of university students in Chongqing who have a correct understanding of NRV% is very small, only 21.2%, and is similar to the findings of some studies [7,23]. NRV% refers to the percentage of certain nutrient content in every 100 g or every food in the recommended daily intake. It may be that the abbreviation of this term is highly technical. Therefore, in our survey, medical students’ understanding of NRV% is much higher than that of students of science, engineering, arts, and sports and may be related to the medical knowledge of medical students [24], who show stronger health literacy than non-medical students and a better understanding of food nutrition labels [25]. In fact, most schools only offer systematic nutrition courses to students in medicine, food and nutrition, and other related majors. Students of other majors rarely have the opportunity to acquire nutrition knowledge in the school’s curriculum. Therefore, nutrition and health courses should be popularized among non-medical students through lectures and elective courses to enhance their health literacy and promote their better understanding of nutrition labels [26]. Moreover, university students have a different understanding of the functional claims of food ingredients in the nutrition labels on prepackaged food.

Paralleling some studies, many people think that the functional claims of food ingredients are only a means of promotion or exaggerated publicity and do not think that it is an introduction of nutritional value [27,28,29]. The functional claim of food ingredients is a kind of allowed and scientific publicity where they are able to claim that a certain nutritional ingredient can maintain the normal growth, development, and normal physiological functions of the human body. Hence, it is also used to convey scientific evidence and knowledge beneficial to health [30] and misconceptions may be related to the lack of nutrition knowledge [31]. It is suggested to strengthen the publicity of the functional claims of food ingredients through mobile phones, the Internet, TV, and other channels to improve the nutritional literacy of students [32]. 

Simultaneously, we found in our survey that university students in Chongqing have many problems in the actual process of choosing the healthiest foods by comparing nutrition labels on prepackaged foods. For example, many university students are not aware of the risks of trans fatty acids. Previous studies have shown that these acids will have negative effects on health, including increasing the risk of hyperlipidemia, heart disease, arterial inflammation, etc. [33,34,35,36,37]. In addition, some university students are unaware of the risks of excessive sodium, which can have adverse effects on the body [38], including increasing the risk of hypertension, coronary heart disease, stroke, and other diseases [39,40,41,42,43]. This is similar to the relevant research reports—despite the differences between theory and practice, the relationship between nutrition knowledge and the use of nutrition labels is inconsistent, sometimes even contradictory [44]. It is suggested that schools can carry out various activities to integrate theory with practice and cultivate students’ ability to understand and use nutrition labels. 

Notably, when asked which prepackaged foods are required to be labeled with food nutrition labels in China, the answers of medical students and non-medical students are not significantly different and both have many misunderstandings. Among them, the situation of mistakenly believing that bottled drinking water needs to be labeled with nutrition labels is more prominent. This may be because Chinese bottled drinking water enterprises have labeled mineral elements, pH, and other information on the outer packaging of products to attract consumers [45], which makes consumers mistakenly believe that they are food nutrition labels. Additionally, some university students think that 40-degree liquor, vinegar, roast chicken, and lettuce need to be labeled with nutrition labels. China’s general rules for nutrition labels on prepackaged food stipulate that fresh food, alcohol with an alcohol content of more than 0.5%, prepared and sold food, packaged drinking water, and prepackaged food with a daily consumption of less than 10 g or 10 mL do not need to be labeled as such [18]. This indicates that schools need to strengthen the education and publicity of nutrition labels on prepackaged food and encourage university students to learn more about the labeling rules of food nutrition labels.

This study also found that there are significant gender differences in the understanding of nutrition labels. Women’s understanding of nutrition labels for prepackaged food is better than men’s, which may be related to women’s greater attention to nutrition and health and better eating habits [46], leading to a higher frequency of using food nutrition labels than men [47]. It is suggested that schools should take measures to promote the use of food nutrition labels by male students and reduce the differences caused by gender. Notably, more than half of the university students reported that they always or often check the food nutrition label when buying prepackaged food, but there was a significant difference in their fields of study. Among them, medical students use the highest proportion of food nutrition labels and science and engineering students use the lowest proportion of nutrition labels, paralleling some studies [17]. This may be related to the fact that medical knowledge of medical students can promote individuals to take healthy behaviors [24]. It is suggested to popularize nutrition knowledge to non-medical students to reduce the difference in the use of food nutrition labels due to differences in majors. 

Meanwhile, our study also shows that there are ethnic differences in the use of nutrition labels on prepackaged food by university students, which is reflected in how ethnic Han students have better cognition of nutrition labels than ethnic minority students, echoing a similar study in Malaysia [48]. This may be due to the lack of access to nutrition knowledge or limited educational opportunities for ethnic minority students [49]. It is suggested to provide more opportunities and conditions for ethnic minority students to popularize nutrition knowledge. Moreover, we also found that education level also affects the use of nutrition labels. In this study, the proportion of senior students and graduate students using nutrition labels is higher than that of junior undergraduates, paralleling a study in Turkey [50]. Simultaneously, compared with other students, the students who have nutrition-related knowledge have more positive performance on the knowledge, attitude, and behavior of nutrition labels than the students who have not, also echoing a study conducted in Portugal [51]. This may be because the senior students received nutrition education earlier than the junior students and have more nutrition knowledge. Therefore, in combination with the above two points, we urge schools to take actions to popularize nutrition knowledge among junior students and publicize food nutrition labels so that students can better cultivate a healthy lifestyle. This study also found that there was a significant difference in the influence of parents’ education on students’ use of food nutrition labels and parents with high education had a better understanding and use of food nutrition labels. This may be because highly educated parents have better nutrition knowledge, which has a positive impact on children [52], paralleling a study in Shanghai, China [21]. Therefore, it is suggested not only to popularize nutrition knowledge to students, but also to popularize nutrition labels to parents.

Our research found that, like some studies, there are still more university students who believe that the nutrition claims on food nutrition labels are untrue and that the label is unnecessary or indifferent [17]. Nutrition labels are a tool to help consumers understand the nutritional composition of food and make correct choices [23,53]. They are an effective way to convey nutrition information to consumers [54]. Frequent use of nutrition labels will reduce the purchase intention of unhealthy food [55] and will have a positive impact on consumers’ diets [56]. Therefore, it is suggested that active intervention measures should be taken to integrate nutrition education into the curriculum of students, to promote university students to acquire more nutritional literacy and healthier eating habits [17,57]. Additionally, we studied the influencing factors of university students’ reading food nutrition labels in Chongqing, China. Following various studies, reasons, such as the labels being not obvious, complex, and incomprehensible, are prominent. These factors affect the observation and correct use of food nutrition labels by university students. Therefore, it is suggested to improve the existing form of nutrition labels [58] and adopt the method of marking on the front of packaging to promote the understanding and use of food nutrition labels by university students [21,59,60].

Although this study achieved some valuable results, because it is a cross-sectional survey, it has certain limitations and cannot explain the temporality and, thus, the degree of causality and specific mechanism brought by long-term follow-up. Moreover, the data in this study are only from Chongqing, China, hence, the deficiencies in representativeness. Meanwhile, due to the impact of COVID-19, this survey was only collected online and the data quality was affected to some extent. There may also be recall bias because the data were collected through self-reporting, which may inevitably introduce some information bias. Nevertheless, as far as we know, this study is the first study on the understanding and use of prepackaged food nutrition labels by university students in Chongqing, China, providing a basis for future research on food nutrition labels.

## 5. Conclusions

Our research shows that university students in Chongqing, China, have a poor understanding and use of the nutrition labels on prepackaged food and some information on the nutrition labels is often misunderstood. More importantly, we found that there were significant differences between different fields of study in the understanding and use of prepackaged food nutrition labels among university students. Compared with other majors, such as science, engineering, and liberal arts, medical students performed better in the understanding and use of nutrition labels. Furthermore, there are still some students who believe that the nutrition label is not important and do not believe in the contents. Therefore, nutrition and health courses should be popularized among non-medical students to enhance their health literacy and narrow the differences between different fields of study. For students in all fields of study, education and publicity of nutrition labels on prepackaged food for university students are needed not only in the classroom but also in daily life for promoting the university students in Chongqing, China, to have a healthier diet and prevent the occurrence of chronic non-communicable diseases.

## Figures and Tables

**Table 1:** Distribution by majors across demographic characteristics.

Factor	Total	Major 1 ^1^	Major 2 ^2^	Major 3 ^3^	Major 4 ^4^	*p*-Value
	Value (n = 1262)	(n = 168)	(n = 420)	(n = 442)	(n = 232)	
Age, mean (SD)	21.825736 (2.4374408)	21.584393 (2.1607858)	21.948252 (2.4059164)	21.658793 (2.5152153)	22.096764 (2.5065471)	0.054
Gender						0.14
Male	630 (49.9%)	83 (49.4%)	194 (46.2%)	224 (50.7%)	129 (55.6%)	
Female	632 (50.1%)	85 (50.6%)	226 (53.8%)	218 (49.3%)	103 (44.4%)	
Ethnicity						<0.001
Han	1020 (80.8%)	152 (90.5%)	338 (80.5%)	320 (72.4%)	210 (90.5%)	
Other	242 (19.2%)	16 (9.5%)	82 (19.5%)	122 (27.6%)	22 (9.5%)	
Father’s education						0.033
Low	434 (34.4%)	67 (39.9%)	141 (33.6%)	153 (34.6%)	73 (31.5%)	
Medium	289 (22.9%)	43 (25.6%)	85 (20.2%)	115 (26.0%)	46 (19.8%)	
High	539 (42.7%)	58 (34.5%)	194 (46.2%)	174 (39.4%)	113 (48.7%)	
Residence						0.19
Rural	642 (50.9%)	94 (56.0%)	201 (47.9%)	235 (53.2%)	112 (48.3%)	
Urban	620 (49.1%)	74 (44.0%)	219 (52.1%)	207 (46.8%)	120 (51.7%)	
BMI						0.66
Normal	832 (65.9%)	116 (69.0%)	276 (65.7%)	287 (64.9%)	153 (65.9%)	
Thinness	31 (2.5%)	6 (3.6%)	9 (2.1%)	12 (2.7%)	4 (1.7%)	
Overweight	396 (31.4%)	45 (26.8%)	133 (31.7%)	143 (32.4%)	75 (32.3%)	
Obese	3 (0.2%)	1 (0.6%)	2 (0.5%)	0 (0.0%)	0 (0.0%)	
Mother’s education						0.027
Low	529 (41.9%)	89 (53.0%)	167 (39.8%)	190 (43.0%)	83 (35.8%)	
Medium	310 (24.6%)	35 (20.8%)	104 (24.8%)	112 (25.3%)	59 (25.4%)	
High	423 (33.5%)	44 (26.2%)	149 (35.5%)	140 (31.7%)	90 (38.8%)	
Income						0.60
<3000	469 (37.2%)	63 (37.5%)	145 (34.5%)	179 (40.5%)	82 (35.3%)	
3000–5000	516 (40.9%)	67 (39.9%)	174 (41.4%)	174 (39.4%)	101 (43.5%)	
5000–10,000	186 (14.7%)	23 (13.7%)	65 (15.5%)	61 (13.8%)	37 (15.9%)	
>10,000	91 (7.2%)	15 (8.9%)	36 (8.6%)	28 (6.3%)	12 (5.2%)	
Dependants						0.066
Parents	619 (49.0%)	90 (53.6%)	213 (50.7%)	216 (48.9%)	100 (43.1%)	
Grandparents and others	643 (51.0%)	78 (46.4%)	207 (49.3%)	226 (51.1%)	132 (56.9%)	
Grade						<0.001
Low	411 (32.6%)	69 (41.1%)	129 (30.7%)	158 (35.7%)	55 (23.7%)	
High	468 (37.1%)	69 (41.1%)	152 (36.2%)	165 (37.3%)	82 (35.3%)	
Postgraduate	383 (30.3%)	30 (17.9%)	139 (33.1%)	119 (26.9%)	95 (40.9%)	
Received courses related to nutrition						<0.001
Yes	658 (52.1%)	157 (93.5%)	213 (50.7%)	191 (43.2%)	97 (41.8%)	
No	604 (47.9%)	11 (6.5%)	207 (49.3%)	251 (56.8%)	135 (58.2%)	

^1^ Major 1 represents medical students, ^2^ Major 2 represents Humanities students, ^3^ Major 3 represents science and engineering students, ^4^ Major 4 represents art and sports students. Differences among students of different majors were examined using the chi-square (χ2) test.

**Table 2:** Comparison of nutrition labels on prepackaged food knowledge level among four different fields of study.

Question	Total	Major 1 ^1^	Major 2 ^2^	Major 3 ^3^	Major 4 ^4^	*p*-Value
	Value (n = 1262)	(n = 168)	(n = 420)	(n = 442)	(n = 232)	
Know the nutrition label on prepackaged food.	892 (70.7%)	150 (89.3%)	300 (71.4%)	283 (64.0%)	159 (68.5%)	<0.001
Frequency of observing food nutrition labels.	934 (74.0%)	140 (83.3%)	301 (71.7%)	317 (71.7%)	176 (75.9%)	0.015
Main contents of the food nutrition label.	370 (29.3%)	64 (38.1%)	112 (26.7%)	127 (28.7%)	67 (28.9%)	0.051
Indicators that must be included in the nutrition label on prepackaged food.	437 (34.6%)	68 (40.5%)	128 (30.5%)	150 (33.9%)	91 (39.2%)	0.046
Prepackaged food with a food nutrition label is required by the state.	249 (19.7%)	37 (22.0%)	90 (21.4%)	76 (17.2%)	46 (19.8%)	0.37
Unusual unit of measurement in the nutrition label.	668 (52.9%)	99 (58.9%)	231 (55.0%)	203 (45.9%)	135 (58.2%)	0.002
Meaning of NRV in the food nutrition label	267 (21.2%)	87 (51.8%)	69 (16.4%)	49 (11.1%)	62 (26.7%)	<0.001
Possible ingredients in the nutritional composition table of “sugar-free coarse fiber biscuits”.	306 (24.2%)	57 (33.9%)	105 (25.0%)	105 (23.8%)	39 (16.8%)	0.001
The packaging of high calcium milk is marked with the meaning of “calcium helps strengthen bones and teeth”.	406 (32.2%)	95 (56.5%)	110 (26.2%)	123 (27.8%)	78 (33.6%)	<0.001
The healthier choice among three food labels.	330 (26.1%)	63 (37.5%)	114 (27.1%)	105 (23.8%)	48 (20.7%)	<0.001
total	269 (21.3%)	67 (39.9%)	80 (19.0%)	69 (15.6%)	53 (22.8%)	<0.001

^1^ Major 1 represents medical students, ^2^ Major 2 represents Humanities students, ^3^ Major 3 represents science and engineering students, ^4^ Major 4 represents art and sports students. Differences among students of different majors were examined using the chi-square (χ2) test. Data presented are the number (percentage) of students who answered the question correctly.

**Table 3:** Association between sociodemographic factors and nutrition labels on prepackaged food knowledge (OR, 95%CI).

Factor	Crude Model	Adjusted Model ^‡^
Residence		
Rural (Ref)		
Urban	0.93 (0.75–1.15)	0.99 (0.81–1.22)
Gender		
Male (Ref)		
Female	1.22 (0.98–1.51)	1.26 (1.03–1.55) **
Ethnicity		
Han (Ref)		
Other	0.08 (0.03–0.19) **	0.10 (0.04–0.25) **
Father’s education		
Low (Ref)		
Medium	1.42 (1.05–1.91)	1.28 (0.94–1.74)
High	1.44 (1.11–1.87) **	1.26 (1.03–1.56)
Mother’s education		
Low (Ref)		
Medium	0.90 (0.67–1.21)	0.78 (0.56–1.08)
High	1.32 (1.04–1.67) **	1.25 (0.82–1.91)
Caregivers		
Parents (Ref)		
Grandparents and others	0.93 (0.75–1.15)	0.93 (0.76–1.13)
BMI		
Normal (Ref)		
Abnormal	0.85 (0.67–1.07)	1.02 (0.82–1.28)
Income		
<3000 (Ref)		
3000–5000	1.25 (0.99–1.58)	0.98 (0.74–1.31)
5000–10,000	0.99 (0.71–1.40)	0.81 (0.55–1.20)
>10,000	0.49 (0.26–0.94) **	0.51 (0.27–0.94) **
Grade		
low (Ref)		
high	1.62 (1.23–2.13) **	1.36 (1.05–1.78) **
Postgraduate	1.46 (1.09–1.95) **	1.35 (1.02–1.78) **
Major		
Medical Science (Ref)		
Humanities	0.48 (0.36–0.63) **	0.62 (0.47–0.81) **
Science and engineering	0.39 (0.29–0.52) **	0.58 (0.43–0.79) **
Art and sports	0.57 (0.42–0.77) **	0.68 (0.50–0.93) **
Received courses related to nutrition		
Yes (Ref)		
No	0.53 (0.42–0.67) **	0.57 (0.45–0.73)

** *p* < 0.05. ^‡^ Adjusted for each other (when exploring the relationship between one demographic factor and understanding and using the nutritional labels on prepackaged food, the others were adjusted).

**Table 4:** Attitudes towards nutritional labels on prepackaged food.

Options	Total	Major 1 ^1^	Major 2 ^2^	Major 3 ^3^	Major 4 ^4^	*p*-Value
	Value (n = 1262)	(n = 168)	(n = 420)	(n = 442)	(n = 232)	
Food nutrition labels shall be marked on food bags.						0.14
1. necessary	907 (71.9%)	126 (75.0%)	304 (72.4%)	309 (69.9%)	168 (72.4%)	
2. unnecessary	51 (4.0%)	4 (2.4%)	20 (4.8%)	24 (5.4%)	3 (1.3%)	
3. indifferent	304 (24.1%)	38 (22.6%)	96 (22.9%)	109 (24.7%)	61 (26.3%)	
Trust the information in the food nutrition label.						0.052
1. trust	258 (20.4%)	43 (25.6%)	84 (20.0%)	89 (20.1%)	42 (18.1%)	
2. partially trust and think the marking is too high	274 (21.7%)	41 (24.4%)	85 (20.2%)	104 (23.5%)	44 (19.0%)	
3. partially trust and think that the mark is too low	235 (18.6%)	34 (20.2%)	84 (20.0%)	72 (16.3%)	45 (19.4%)	
4. partially trust and think the marking is incomplete	283 (22.4%)	34 (20.2%)	99 (23.6%)	104 (23.5%)	46 (19.8%)	
5. do not trust	212 (16.8%)	16 (9.5%)	68 (16.2%)	73 (16.5%)	55 (23.7%)	
The main purpose of observing the nutrition label.						<0.001
1. prevent excessive intake	251 (19.9%)	56 (33.3%)	73 (17.4%)	75 (17.0%)	47 (20.3%)	
2. judge the health of food	296 (23.5%)	54 (32.1%)	89 (21.2%)	102 (23.1%)	51 (22.0%)	
3. calculate the total energy of food	271 (21.5%)	37 (22.0%)	101 (24.0%)	86 (19.5%)	47 (20.3%)	
4. just browse	444 (35.2%)	21 (12.5%)	157 (37.4%)	179 (40.5%)	87 (37.5%)	
Main reasons affecting reading food nutrition labels.						
1. The label is too troublesome and a waste of time	409 (32.4%)	45 (26.8%)	138 (32.9%)	145 (32.8%)	81 (34.9%)	0.37
2. The label is not obvious, the word is too small or cannot be found.	423 (33.5%)	53 (31.5%)	154 (36.7%)	131 (29.6%)	85 (36.6%)	0.10
3. The label is complex and difficult to understand.	455 (36.1%)	51 (30.4%)	147 (35.0%)	163 (36.9%)	94 (40.5%)	0.19
4. Do not trust the label.	470 (37.2%)	55 (32.7%)	148 (35.2%)	172 (38.9%)	95 (40.9%)	0.25
5. Do not care about nutrition, taste and brand are more important.	480 (38.0%)	53 (31.5%)	166 (39.5%)	168 (38.0%)	93 (40.1%)	0.28
6. Do not know the nutrition label.	411 (32.6%)	41 (24.4%)	144 (34.3%)	137 (31.0%)	89 (38.4%)	0.021
7. Look at the nutrition label every time.	143 (11.3%)	38 (22.6%)	43 (10.2%)	51 (11.5%)	11 (4.7%)	<0.001

^1^ Major 1 represents medical students, ^2^ Major 2 represents Humanities students, ^3^ Major 3 represents science and engineering students, ^4^ Major 4 represents art and sports students. Differences among students of different majors were examined using the chi-square (χ2) test.

## Data Availability

Not applicable.

## Supplementary Materials

The following supporting information can be downloaded at: https://www.mdpi.com/article/10.3390/nu14194189/s1, Figure S1: Nutrition label of Sprite in China; Table S1: Questionnaire.
