# Supplementary material for: Understanding and Use of Nutrition Labels of Prepackaged Food by University Students: A Cross-Sectional Study in Chongqing, China
Source: Nutrients. 2022 Oct 8;14(19):4189. doi: 10.3390/nu14194189 (PMC9573194; doi:10.3390/nu14194189)

Supplementary Table S1. Questionnaire.

Questionnaire No: Date of investigation:

Survey on the understanding and use of nutrition labels for pre-packaged food among college students in Chongqing

Dear students,

In order to evaluate the understanding and use of nutrition labels for prepackaged food among university students in Chongqing, we now conduct this survey. Prepackaged food refers to foods commonly found in stores and supermarkets that have been uniformly packaged and labeled. This questionnaire is anonymous, please fill in according to the actual situation, and the recovered data are only used for scientific research. We will keep all your answers confidential, please feel free to fill in, thank you very much for your support and cooperation.

1. Gender
a. Male
b. Female

2. Date of birth: _______

3. University name: _______

4. Grade
a. First year undergraduate
b. Sophomore
c. Third year undergraduate
d. Fourth year undergraduate
e. Fifth year undergraduate
f. First year postgraduate
g. Second year postgraduate
h. Third year postgraduate

5. Category of major
a. Medicine
b. Humanities
c. Science and engineering
d. Art and sports
e. Other

6. Have you studied food or nutrition related courses?
a. Yes
b. No

7. Height: _______

8. Weight: ______

9. Nationality
a. Han
b. Ethnic minorities: _______

10. Long term residence of family
a. City
b. Rural

11. Native place:_______

12. What is the father's educational level?
a. Primary school and below
b. Junior high school, technical secondary school
c. High school, vocational high school
d. College
e. Undergraduate
f. Postgraduate

13. What is the mother's educational level?
a. Primary school and below
b. Junior high school, technical secondary school
c. High school, vocational high school
d. College
e. Undergraduate
f. Postgraduate

14. Who is the long-term caregiver before adulthood?
a. Parents
b. Grandparents
c. Others: _______

15. What is the average monthly income of each household?
a. Less than RMB 2000 per person
b. RMB 2000-3000 per person
c. RMB 3000-5000 per person
d. RMB 5000-10000 per person
e. More than RMB 10000 per person

16. Do you know the nutrition label of prepackaged food?

a. Yes
b. No

17. From which channels do you get knowledge about nutrition labels? (Multiple choices)
a. TV, radio
b. Books, newspapers, magazines
c. Instant chat tools such as WeChat and QQ
d. Tiktok, Kwai and other short video software
e. Courses and expert lectures
f. Explanation by the health care product salesperson
g. Others: ________
h. I don't know the nutrition label

18. How often do you observe nutrition labels when purchasing prepackaged food?
a. Every time
b. Often
c. Occasionally
d. Basically no observation
e. Never observed

19. What does the food nutrition label mainly include?
a. Food name, ingredients and eating method
b. Food production date, shelf life and storage conditions
c. Food nutrition composition table, nutrition claim and nutrition function claim
d. Food production batch number, name and address of the manufacturer
e. I don't know

20. What are the main reasons that affect your view of nutrition labels? (Multiple choices)
a. It's too troublesome to read the labels. It's a waste of time
b. The label is not obvious, the word is too small or cannot be found
c. The label is complex and difficult to understand
d. Do not believe the label
e. Don't care about nutrition, taste and brand are more important
f. I don't know the nutrition label
g. No effect. Look at the nutrition label every time

21. What indicators must be included in the nutrition label of prepackaged food?
a. Amino acid, protein, fat, vitamins, minerals
b. Energy, protein, fat, carbohydrate, sodium
c. Energy, amino acid, fat, carbohydrate, edible essence
d. Water, starch, minerals, vitamins, sodium glutamate, spices
e. Water, fat, starch, sodium, edible essence

22. Do you think food bags need to be labeled with food nutrition labels?

a. Necessary
b. Not necessary
c. It doesn't matter

23. Do you believe the information in the food nutrition label on the food bag?
a. Trust
b. Some believe that some nutritional ingredients are labeled too high
c. Some believe that some nutrients are labeled too low
d. Some believe that the label of nutrient composition is incomplete
e. Do not trust

24. What is the main purpose of reading the nutrition label?
a. Prevent some nutrients from exceeding the standard
b. Judge whether the food is healthy
c. Calculate total energy intake
d. Just browse
e. Do not observe labels

25. What prepackaged foods are required by the state to be labeled with food nutrition labels? (Multiple choices)
a. Bottled purified water
b. Sprite
c. 40-degree liquor
d. Lettuce
e. Bagged sugar free biscuits
f. Instant noodles
g. Fresh Roast Chicken
h. Vinegar

26. Common measurement units in the nutrition ingredient label do not include
a. Per 30g
b. Per 100g
c. 150g per bag
d. Per 100 ml
e. Every 500g

27. What does the NRV in the following label mean?

Nutrition Table

Items	per 100g	NRV%
Protein	20g	33%

a. The protein in 100g of this food can meet 33% of the daily needs of the human body
b. 33% of the protein in 100g of this food can meet daily needs

c. 33% of the 100 grams of this food consists of protein

d. 33% of the protein in 100g of this food can be absorbed by the human body

e. I don't know

28. What may be included in the nutrition label of sugar free coarse fiber biscuits?

a. Carbohydrate 56g, protein 0g

b. Carbohydrate 0g, protein 10g

c. Carbohydrate 56g, dietary fiber 10g

d. Carbohydrate 0g, dietary fiber 10g

e. Protein 0g, dietary fiber 0g

29. What is the purpose of the label "Calcium helps strengthen bones and teeth" on the package of high calcium milk?

a. False propaganda

b. Induced consumption

c. Publicize nutritional functions

d. Beautify packaging

e. I don't know

30. Which of the three foods shown below has higher nutritional value?

Nutrition Table

Items	per 100g	NRV%
Energy	2209KJ	26%
Fat	4.8g	8%
-trans fatty acids	4g	
Calcium	250mg	32%
Vitamin D	2.0μg	40%

Nutrition Table

Items	per 100g	NRV%
Energy	1513KJ	18%
Fat	5.2g	9%
Carbohydrates	21g	7%
Vitamin C	23μg	23%

Nutrition Table

Items	per 100g	NRV%
Energy	1513KJ	16%
Fat	2.4g	4%
Sodium	1815mg	91%
Dietary fiber	1.8g	8%

a. First

b. Second

c. Third

d. All the same

e. I don't know

Supplementary Figure S1. Nutrition label of Sprite in China.

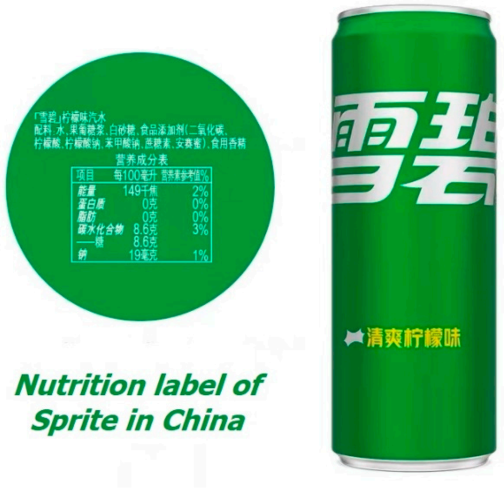

Supplement: Supplementary file 1 [file nutrients-14-04189-s001.zip › nutrients-1910580-supplementary.pdf]
